# Supplementary figures and images for: A close relative of the Amazon river dolphin in marine deposits: a new Iniidae from the late Miocene of Angola
Source: PeerJ. 2018 Sep 12;6:e5556. doi: 10.7717/peerj.5556 (PMC6139015; doi:10.7717/peerj.5556)

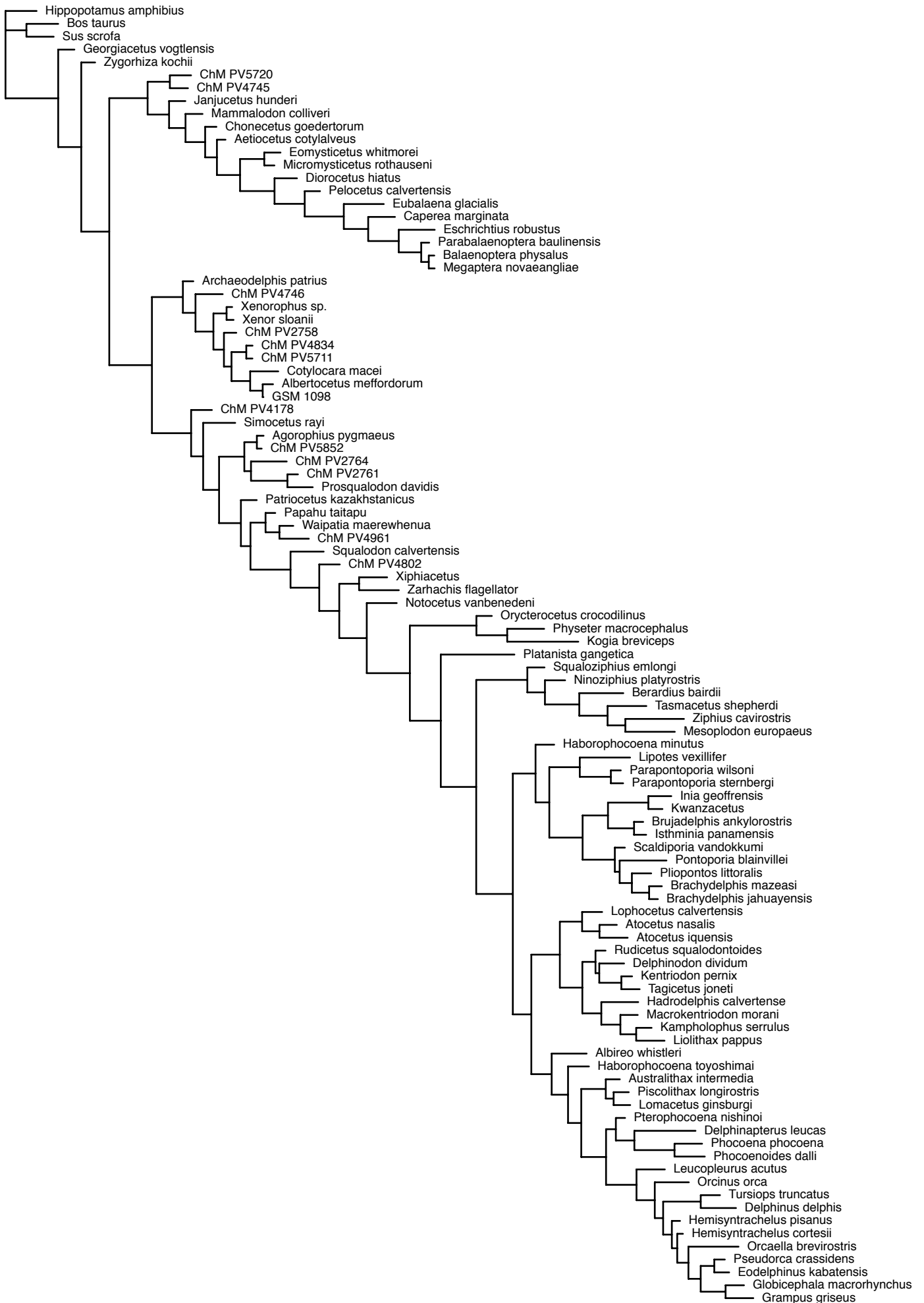

Supplement: Supplemental Information 1 [file peerj-06-5556-s001.pdf]
